# Expression of Iron-Related Proteins Differentiate Non-Cancerous and Cancerous Breast Tumors

**DOI:** 10.3390/ijms18020410

**Published:** 2017-02-14

**Authors:** Sara Pizzamiglio, Maida De Bortoli, Elena Taverna, Michele Signore, Silvia Veneroni, William Chi-shing Cho, Rosaria Orlandi, Paolo Verderio, Italia Bongarzone

**Affiliations:** 1 Unit of Medical Statistics, Biometry and Bioinformatics, Fondazione IRCCS Istituto Nazionale dei Tumori, 20133 Milan, Italy; sara.pizzamiglio@istitutotumori.mi.it (S.P.); paolo.verderio@istitutotumori.mi.it (P.V.); 2 Proteomics Laboratory, Fondazione IRCCS Istituto Nazionale dei Tumori, 20133 Milan, Italy; maida.debortoli@istitutotumori.mi.it (M.D.B.); elena.taverna@istitutotumori.mi.it (E.T.); 3 Department of Hematology, Oncology and Molecular Medicine, Istituto Superiore di Sanità, 00161 Rome, Italy; michele.signore@iss.it; 4 DOSMM, Fondazione IRCCS Istituto Nazionale dei Tumori, 20133 Milan, Italy; silvia.veneroni@istitutotumori.mi.it; 5 Department of Clinical Oncology, Queen Elizabeth Hospital, Hong Kong, China; chocs@ha.org.hk; 6 Molecular Targets Unit, Fondazione IRCCS Istituto Nazionale dei Tumouri, 20133 Milan, Italy; rosaria.orlandi@istitutotumori.mi.it

**Keywords:** Iron, metabolism, breast cancer, hepcidin, ferroportin, transferrin receptor (TFR), STAT5, reverse phase protein array, iron chelators

## Abstract

We have previously reported hepcidin and ferritin increases in the plasma of breast cancer patients, but not in patients with benign breast disease. We hypothesized that these differences in systemic iron homeostasis may reflect alterations in different iron-related proteins also play a key biochemical and regulatory role in breast cancer. Thus, here we explored the expression of a bundle of molecules involved in both iron homeostasis and tumorigenesis in tissue samples. Enzyme-linked immunosorbent assay (ELISA) or reverse-phase protein array (RPPA), were used to measure the expression of 20 proteins linked to iron processes in 24 non-cancerous, and 56 cancerous, breast tumors. We found that cancerous tissues had higher level of hepcidin than benign lesions (*p* = 0.012). The univariate analysis of RPPA data highlighted the following seven proteins differentially expressed between non-cancerous and cancerous breast tissue: signal transducer and transcriptional activator 5 (STAT5), signal transducer and activator of transcription 3 (STAT3), bone morphogenetic protein 6 (BMP6), cluster of differentiation 74 (CD74), transferrin receptor (TFRC), inhibin alpha (INHA), and STAT5_pY694. These findings were confirmed for STAT5, STAT3, BMP6, CD74 and INHA when adjusting for age. The multivariate statistical analysis indicated an iron-related 10-protein panel effective in separating non-cancerous from cancerous lesions including STAT5, STAT5_pY694, myeloid differentiation factor 88 (MYD88), CD74, iron exporter ferroportin (FPN), high mobility group box 1 (HMGB1), STAT3_pS727, TFRC, ferritin heavy chain (FTH), and ferritin light chain (FTL). Our results showed an association between some iron-related proteins and the type of tumor tissue, which may provide insight in strategies for using iron chelators to treat breast cancer.

## 1. Introduction

Iron is an essential trace element for both physiological cellular functions and neoplastic cell growth [1]. Iron is a cofactor for key enzymes of DNA duplication, repair, and epigenetics. Continued multiplication, growth, and survival of malignant cells require high iron metabolism [1].

Cancer cells show an increase in transferrin receptor (TFR), which is responsible for uptake and regulation of higher levels of intracellular iron levels. Notably, breast cancer shows both elevated levels of TFR and decreased levels of the iron exporter ferroportin 1 (FPN1), both in breast cancer tissue and cancer cell lines with a higher malignancy potential, denoting an iron-reserving phenotype, compatible with their increased proliferative status [2].

A higher percentage of breast carcinomas, compared to control mastectomy samples, present iron accumulation in stromal inflammatory cells [3]. Tumor-associated macrophages that secrete ferritin and senescent erythrocytes in the microenviroment in breast cancer contribute by providing iron to tumor cells. The levels of ferritin light chain are about six-fold higher than in surrounding benign breast tissue and this increase correlates with greater epithelial cell proliferation, histopathological dedifferentiation, shorter survival rates, and chemotherapeutic resistance [4]. Mertens et al. have recently demonstrated that macrophages and their iron-release phenotype would favor tumor progression and metastasis [5]. 

Hepcidin exerts an iron homeostatic control inducing iron sequestration in macrophages and the resulting stimulation of ferritin synthesis virtually explains the high serum ferritin observed in inflammation and in some types of cancers, and the high correlation between serum hepcidin and ferritin levels have been recently reported [6]. Serum ferritin has been associated with breast cancer risk and recurrence [7,8].

Iron metabolism, hepcidin increase, inflammation, and anemia in breast cancer are interconnected processes. Iron loading, inflammation, and interleukin-6 (IL-6) increase the production of hepcidin [9], determining anemia, increased erythropoietin, or hypoxia, which stimulates bone marrow erythropoiesis. In contrast, erythropoietin and other erythropoiesis-stimulating agents probably decrease hepcidin expression [10,11].

We have observed that hepcidin and ferritin light chain levels in plasma may predict malignant and benign disease with respect to healthy controls [6]. These findings corroborate the concept that altered systemic homeostasis is a typical feature of breast cancer patients. In a following study, we observed that upregulated hepcidin in the plasma of breast cancer patients is not directly associated with IL-6 augmentation [12].

Investigating the molecular mechanisms underlying the most frequent genetic disorder and signaling pathways involved in cancer a large number of proteins involved in iron metabolism has emerged. Specifically, those related to AKT, ERK, JNK, p38, STAT3, TGF-β, and WNT functions [13] also play roles in cellular mechanisms that include apoptosis, autophagy, cellular migration, stem cell proliferation, angiogenesis, immune cell modulation, and tumorigenesis. 

Starting from previous considerations, and specifically, from the evidence that hepcidin and ferritin increase in plasma of breast cancer patients but not in patients with benign conditions, we speculated to find alterations in the expression of factors reflecting dysfunctional iron metabolism in cancerous conditions. In this study we identified a panel of proteins involved in iron-related processes that are differentially expressed in cancerous tumor samples compared with non-cancerous tumor samples. These determinations could be conducted to predict the potential effectiveness of iron chelators used as therapeutic agents in patients [14].

Reverse-phase protein array (RPPA) is recently emerged as a powerful high-throughput approach for targeted proteomics [15]. Here we used enzyme-linked immunosorbent assay (ELISA) and RPPA to determine the tissue expression of a panel of iron-related protein. As a major advantage, RPPA allows to assess target protein expression quantitatively in large sample sets while requiring only a very low amount of biological sample making this platform attractive for the analysis of clinical materials. Quantitative protein expression data was generated measuring the expression of 17 iron-related proteins in 56 malignant tumors and 24 benign tumors. Of relevance, our statistical analysis suggested that a subset of the selected iron-related proteins was differentially expressed in cancerous vs. non-cancerous breast lesions. This study investigates some cancer-specific dysfunctions in iron metabolic pathways, which implementing previous results obtained by us and others on the key role played by iron metabolism in breast cancer and provide new possible tools for treatment.

## 2. Results

### 2.1. Hepcidin, Interleukin-6 and Erythropoietin Levels in Breast Cancerous and Non-Cancerous Tumor Tissues

Hepcidin, IL-6 and erythropoietin (EPO) expression were determined by ELISA in the 56 cancerous and 24 non-cancerous breast tissues. Table 1 shows the descriptive statistics for hepcidin, IL-6 and EPO according to the status of the tumor tissue. 

**Figure 1:**
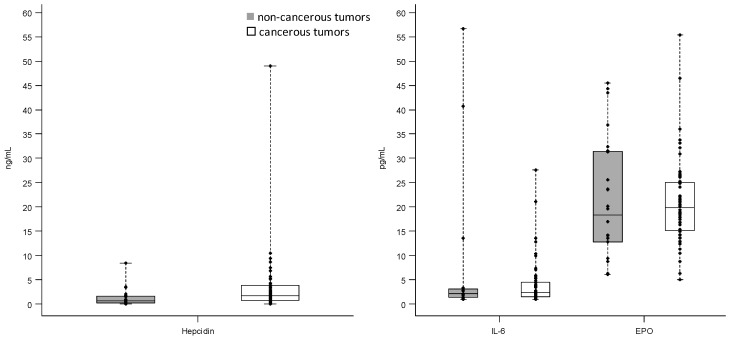
Distribution of hepcidin, interleukin-6 (IL-6), and erythropoietin (EPO) levels measured by enzyme-linked immunosorbent assay (ELISA) in breast cancerous and non-cancerous tumor tissues; Each box indicates the 25th and 75th percentiles. The horizontal line inside the box indicates the median, and the whiskers indicate the extreme measured values; each individual value is represented by a dot.

As shown by the interquartile range (IQR) values, the variability of IL-6 and hepcidin was higher in breast cancerous tissue, whereas the variability of EPO was higher in non-cancerous one. The box plots reported in Figure 1 describe the distribution of hepcidin, IL-6, and EPO levels measured by ELISA according to the tumor lesion.

According to the non-parametric tests of Wilcoxon–Mann–Whitney, only the levels of hepcidin were statistically different between cancerous and non-cancerous tissues (*p* = 0.012). However, no significant association was found between the protein levels and the tumor lesion (cancerous vs. non-cancerous) adjusting for age in logistic regression model. 

### 2.2. Comparison of 17 Iron-Related Proteins Measured by Reverse-Phase Protein Array between Non-Cancerous and Cancerous Tumors 

**Figure 2:**
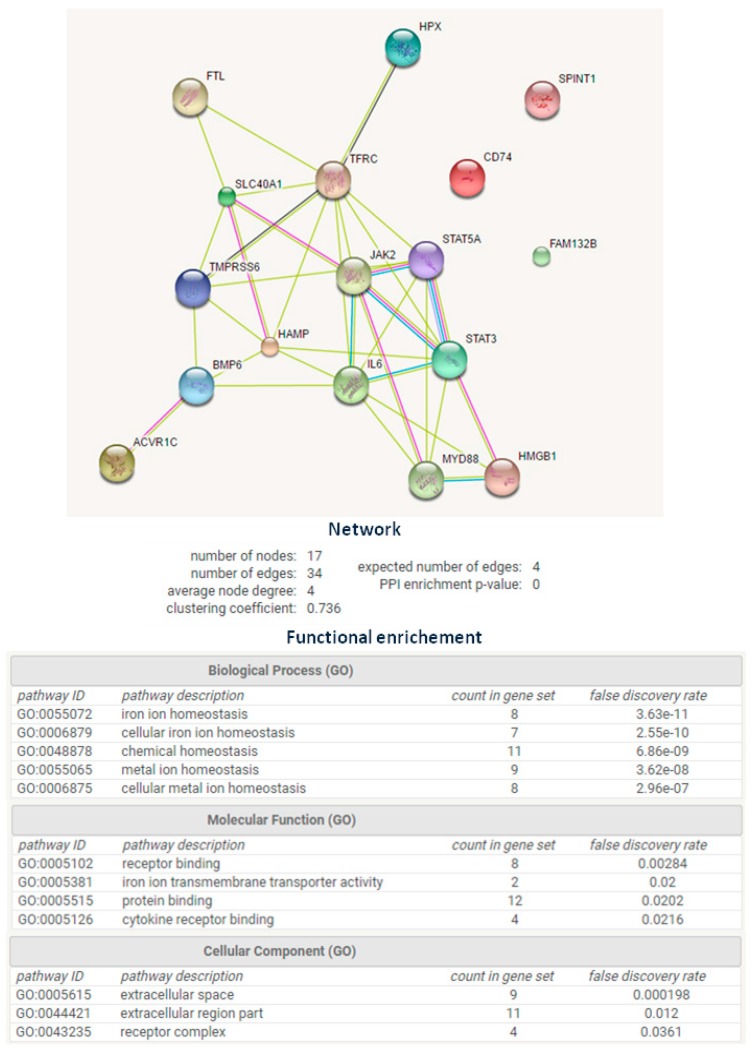
Network generated by the 17 selected proteins according to STRING 9.0 Web software; the network formed by INHA, HMGB1, CD74, SLC40A1/TFRC, SPINT1, FPN, MYD88, JAK2, STAT3, FTL, STAT5, BMP6, TMPRSS6, HPX, IL-6, HAMP, and ERFE/FAM132B (Q8NER5, P09429, P04233, P02786, O43278, Q9NP59, Q99836, O60674, P40763, P02792, P42229, P22004, Q8IU80, P02790, P05231, P81172, and Q4G0M1) supported the membership of selected proteins to iron homeostasis biological process (FDR = 3.63 × 10^−11^). FDR*:* False Discovery Rate; GO: Gene ontology.

The expression of 35 iron-linked proteins and phosphoproteins were assessed with a panel of antibodies (data not shown). A final set of 17 antibodies was chosen for read-outs based on Western blot experiments, quality control of slides, correlation between technical replicates, and evidence of the protein being relevant for iron biology (Figure 2). Specifically, INHA, HMGB1, TFRC, FPN, MYD88, JAK2, STAT3, FTL, STAT5, BMP6, TMPRSS6, and HPX generated a network based on their co-expression and homologies at gene and protein levels, as assessed by STRING 9.0 software [16] (Figure 2). 

In additional to this panel of proteins we add FAM132B/ERFE/erythroferrone [11], CD74, and Kunitz-type serine protease inhibitor (SPINT) [17]. FAM132B/ERFE/erythroferrone, is a new iron-related protein not yet included in STRING 9.0 database and it was inserted because it inhibits the action of hepcidin, and so increases the amount of iron availability. Cluster of differentiation 74 (CD74) was selected as a marker of monocyte/macrophage infiltration, which are pivotal players in iron metabolism [18] and SPINT was included because of its involvement in cellular response to bone BMP6, a factor extremely important in iron metabolism processes [17]. The few data available on its expression in tissue made attractive its determination. 

We quantified the 17 proteins by RPPA, 14 proteins were reported in Figure 2, plus two phosphorylated isoforms, STAT3_pS727 and STAT5_pY694, to determine the amount of STAT3 and STAT5 activated proteins. We also measured the levels of ferritin heavy chain (FTH) because it was reported increase in the blood of breast cancer patients [19]. 

#### 2.2.1. Univariate Analysis

Table 2 presents the descriptive statistics of the 17 iron-related proteins measured by RPPA according to the breast tumor lesion.

**Figure 3:**
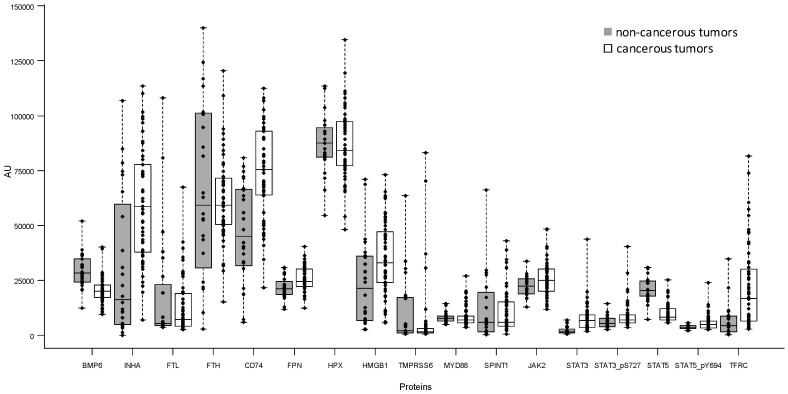
Box plot of the 17 iron-related proteins measured by RPPA (arbitrary units, AU) in breast cancerous and non-cancerous tumor tissues; each individual value is represented by a dot.

Figure 3 presents the box plot of the 17 iron-related proteins measured by RPPA. According to the Bonferroni adjusted *p*-value the Wilcoxon–Mann–Whitney test identified seven proteins with a statistically different expression between non-cancerous and cancerous breast lesion (STAT5 *p* < 0.0001; STAT3 *p* < 0.0001; BMP6 *p* = 0.0002; CD74 *p* = 0.0003; TFRC *p* = 0.0012; INHA *p* = 0.0051; STAT5_pY694 *p* = 0.034). STAT5 and BMP6 are more expressed in non-cancerous tissue whereas the other five significant proteins presented elevated levels in cancerous ones. When we evaluated the association between the proteins expression and the tumor lesion (breast cancerous vs. non-cancerous) in logistic regression model adjusting for age, the following five proteins resulted statistically significant according to the Bonferroni *p*-value: STAT5 (*p* < 0.0001), BMP6 (*p* = 0.0051), CD74 (*p* = 0.0034), INHA (*p* = 0.0085), and STAT3 (*p* = 0.0085).

Of note, all seven proteins with statistically different expressions between non-cancerous and cancerous breast lesion also showed the same qualitative trend (increase or decrease) in Western blot experiments (Figure S1). 

#### 2.2.2. Multivariate Analysis

**Figure 4:**
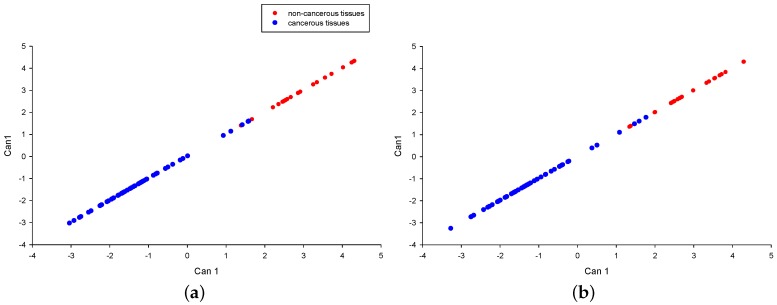
Linear discriminate analysis. (**a**) Linear discriminate analysis of all the 17 iron-related proteins measured by RPPA technology; (**b**) linear discriminate analysis of the top 10 selected proteins; the red and blue dots indicate non-cancerous and cancerous tissues, respectively; Can1: discriminate function.

In Figure 4a, the first canonical correlation is presented (Can1, discriminate function), obtained by applying the liner discriminate analysis to all the 17 proteins. From this multivariate analysis emerges a clear ability of the joint combination of all the protein in discriminating benign from malignant tissues with an overall value of squared Mahalanobis distance (SMD) between groups equal to 17.54. There are only four subjects not correctly allocated (misclassified samples). 

#### 2.2.3. Selection of Relevant Proteins for Discriminate Between Non-Cancerous and Cancerous Tumors

The SMD computed by exuding, one by one, each of the 17 considered proteins was used to assess the contribution to the discriminatory capability of each protein. The resulting top 10 proteins were: STAT5, STAT5_pY694, MYD88, CD74, FPN, HMGB1, STAT3_pS727, TFRC, FTH, and FTL. The first canonical correlation obtained by considering the top 10 proteins is depicted in Figure 4b. This reduction of the number of proteins allows preserving the same discriminatory capability observed by considering the 17 proteins panel; specifically, a similar value of SMD between groups (SMD = 16.3), as well as the same four misclassified subjects were observed. Of note, similar results (data not shown) were obtained by including into the multivariate analyses the variable age. In addition, in order to test 10 panel proteins, we performed leave-one-out cross-validation on our dataset and the resulting number of misclassified patients was equal to seven, both with and without age. 

#### 2.2.4. Exploratory Evaluation of the Expression of TFRC, FPN, FTL, and FTH with the Clinicopathological Data

We performed the comparisons of the expression of TFRC, FPN, FTL, and FTH according to the estrogen receptor (ER) status in malignant tissues. None of the four proteins resulted statistically significant according to the Wilcoxon–Mann–Whitney test. 

## 3. Discussion

Glucose metabolism is an attractive target for cancer therapy given the clinical observation that many tumors exhibit a significant increase in glucose uptake compared with adjacent normal tissue or benign counterpart [20]. Iron represents a micronutrient that is required for metalation of the proteins needed for glucose oxidation and glucose sensing [21]. A large body of data indicates that breast tumors undergo broad changes in iron metabolism [3]. These alterations would create metabolic dependencies of tumors. For this reason, enzymes involved in iron homeostasis could represent prognostic indicators, as well as valid pharmacological targets in breast cancer therapy. Iron chelators are largely used in clinic could be used to treat patients to prevent disease progression [22].

It is remarkable that iron homeostatic pathways are tightly linked to inflammatory stressors. Thus, inflammation causes significant upregulation of hepcidin, largely through IL-6, and also results in large increases in serum ferritin levels [23]. Additionally, increases of ferritin either reflecting excess iron stores, or reflecting inflammation, or both, are observed in breast cancer. Clinically, circulating ferritin is used as a biomarker of inflammation or as an indicator of the body’s iron stores. In the context of breast cancer, ferritin is elevated in patients’ sera, as well as in tumor tissue, and this elevation correlates with poor clinical outcomes and an advanced histological grade [4].

Hepcidin levels in plasma of breast cancer patients are increased and these increases were not observed in non-cancerous breast conditions [6].

The goal of this study was to assess whether a small number of proteins interlinked with iron homeostatic pathways are differentially expressed in breast cancerous lesions compared with breast non-cancerous lesions. 

Using the STRING 9.1 database, which provides known and predicted protein–protein associations, we extrapolated a subset of proteins connected with morphogenesis, inflammation, erythropoiesis, and with physiological relevance in iron trafficking and metabolism remodeling (Figure 2). We analysed the expression of this panel of proteins using ELISA or a miniaturized multiple protein analysis platforms such as RPPA to parallel profiling of multiple protein markers [15,24].

We found that cancer tissues have greater levels of hepcidin than benign lesions (*p* = 0.012) even if this statistical significance was not confirmed by considering the covariate age. This, in our study, is not accompanied by a higher expression of IL-6 and EPO making more complicated the determination of interplay between mediators of iron homeostasis, inflammation, and erythropoiesis. This results are coherent with our previous ones supporting the lack of a relationship between the plasma levels of hepcidin and both IL-6 and EPO [6].

However, the univariate analysis of RPPA data highlighted the following seven proteins differentially expressed between non-cancerous and cancerous tumors: STAT5 *p* < 0.0001; STAT3 *p* < 0.0001; BMP6 *p* = 0.0002; CD74 *p* = 0.0003; TFRC *p* = 0.0012; INHA *p* = 0.0051, and STAT5_pY694 *p* = 0.034. These findings were confirmed for five of them by taking into consideration also the covariate age (STAT5 (*p* < 0.0001), BMP6 (*p* = 0.0051), CD74 (*p* = 0.0034), INHA (*p* = 0.0085), and STAT3 (*p* = 0.0085).

The morphogenetic BMP signaling (BMP6 and INHA), the pro-inflammatory JAK/STAT pathway and CD74, a pro-inflammatory component in terms of infiltrating macrophages, resulted in discriminating between cancer and non-cancer samples. Transferrin receptor expression is increased in breast cancer tissues confirming previous observations. It has been seen implicated in promoting the growth of endocrine resistant phenotypes within ER+/luminal-like breast cancer [25]; however, no correlation with ER status was observed in the present study. Its over-expression could be related due to the presence of hypoxia and iron requirements in cancer cells. A hypoxic microenvironment is a hallmark for solid tumors [26]. Generally, low levels of iron may stimulate HIF1α activation and consequently promote angiogenesis and progression [27]. 

STAT5 results to be the protein providing the higher contribution in discriminating benign and malignant tissues in terms of both espression and phosphorylation. STAT5 is important in cytokine-mediated immune responses. Malignant tissues have more activated STAT5, supporting the interpretation that EPO not being different in benign and malignant samples could be only one factor activating the STAT5 pathway, opening the possibility to others factors, such as prolactin, and others factors may activate the pathway [28]. Analogously STAT3 potentially regulated by cytokines like IL-6 was a discriminating feature being higher in malignant tissues. Additionally, in this case IL-6 could be only one cytokine able to activate STAT3 signaling; for example leptin could increase the level of STAT3 phosphorylation [29]. 

BMP6 belongs to the TGF-β superfamily. Recent studies indicated that BMP6 is the most important physiologic regulator of hepcidin expression in response to iron overload [30]. We observed that BMP6 was downregulated in breast cancer. 

We integrated quantitative information with a multivariate statistical analysis in order to extract further relevant information. From this analysis, it was identified an iron-related 10-protein panel effective in separating benign lesions from malignant one. 

This panel includes the following proteins: STAT5, STAT5_pY694, MYD88, CD74, FPN, HMGB1, STAT3_pS727, TFRC, FTH, and FTL. Among these, four proteins (STAT5, STAT5_pY694, CD74, TFRC) also presented a significant, statistically different expression between benign and malignant tissue in the univariate analysis. 

Interestingly, perturbations in ferritin levels are associated with breast cancer progression toward a more advanced malignant phenotype [31]. Increases of FTL were largely documented in breast cancer with respect to normal tissue; here we document differences with respect to benign conditions. FTL increase in cancer lesions was potentially due to the contribution of cancer-associated macrophages secreting ferritin, particularly in response to pro-inflammatory cytokines, but also that extracellular ferritin stimulates the proliferation of breast cancer cells [4]. This favors the shift of tumor-associated macrophages vs. alternatively activated macrophages (M2)-like phenotype that enhances iron release and availability in tumor microenvironment. An M2 macrophage polarization was also compatible with the observed CD74 enriched expression pattern (Figure 3) and the elevated levels of macrophage migration inhibitory factor (MIF) cytokine in the cancer specimens (data not shown). Our results on CD74 are in agreement with data reported in the literature [32].

In breast cancer cells, it has been reported a lowered level of FPN1 causing iron accumulation in cancer cells [33]. We do not observe differences in our study comparing benign and malignant conditions. This finding could be explained by the high ferroportin expression in adipocytes major components in non-cancerous tumor tissue [34]. On the other hand, lymphocytes and macrophages could contribute to FPN positivity in breast carcinoma samples, increasing the total expression due to epithelial components [2].

The results suggest the existence of an iron-related set of proteins that, when jointly considered, are able to discriminate benign and malignant breast lesions. Therefore, these results are relevant as they may have potential for clinical decisions regarding precision medicine strategies for using iron chelators to treat breast cancer patients. 

## 4. Materials and Methods 

### 4.1. Samples Characteristics

Cancerous breast tissues were consecutively collected during the period 1990–1993 at Fondazione IRCCS Istituto Nazionale Tumori of Milan. Table S1 reports the available clinical-pathological characteristics of the 56 patients. The 24 non-cancerous tumors were collected during the period 2002–2013 and were classified as follows: 10 fibroadenoma, 10 hyperplasia, two papillomas, and two benign phyllodes tumors. The median age at surgery was greater in patients with malignant tumors (median age: 57 years, range 29–82 years) with respect to patients with benign tumors (median age 44 years, range 20–65 years). The study was conducted in accordance with the Declaration of Helsinki, and the protocol was approved by the Ethics Committee with recorded code number D275740.

Proper procedures for specimen collection and storage were adopted. Frozen tissues were banked and have been maintained continuously frozen at −80 C. Western blots were post-stained with SYPRO^®^ Ruby Protein Blot Stain (Invitrogen Life Science Technologies, Carlsbad, CA, USA) to ensure that samples have been properly stored and high quality protein patterns was reported (Figure S2). Of relevance is that the quality of proteins was comparable to that of non-cancerous samples.

### 4.2. Enzyme-Linked Immunosorbent Assay

Tissue hepcidin-25 was evaluated by competitive ELISA (Human Hepcidin-25 EIA Kit; Bachem, Bubendorf, Switzerland) using a nine-point serial two-fold dilution standard curve following the manufacturer’s recommendation. The reported normal range for this assay is 0.02–25 ng/mL, and both the calculated intra- and inter-assay variations are below 10%. Tissue levels of IL-6 were determined using the IL-6 High Sensitivity Human ELISA Kit (Abcam, Cambridge, UK); the reported detection range for this assay is 1.56–50 pg/mL. Tissue levels of EPO were determined using the ELISA Kit from Cloud-Clone Corp (Houston, TX, USA); the detection range for this assay is 31.25–2000 pg/mL. Tests were performed in duplicate.

### 4.3. Reverse-Phase Protein Array 

Reverse-phase protein array was performed as previously described [26]. Samples were lysed using Tissue Protein Extraction Reagent (Thermo Scientific, Waltham, MA, USA) and diluted up to 0.5 mg/mL with Novex Tris-Glycine SDS Sample Buffer 2X (Invitrogen Corporation, Carlsbad, CA, USA). Each lysate was spotted in a two-fold 5 point dilution curve onto nitrocellulose-coated microscope slides (Grace Biolabs, Bend, OR, USA) using an Aushon Arrayer 2470 (Aushon Biosystems, Billerica, MA, USA). Slides were incubated with a single pre-validated primary antibody using DAKO Autostainer Plus (DAKO Corporation, Glostrup, Denmark). Slides underwent signal amplification (CSA kit, DAKO) and staining with streptavidin-conjugated IRDye680LT (LI-COR Biosciences, Lincoln, NE, USA). Stained slides were scanned on a Tecan Power Scanner (Männedorf, Switzerland) equipped with a customized emission filter to increase efficiency in collection of IRDye680LT fluorescence. Image analysis for spot recognition, quantification and normalization was carried out using MicroVigene 5.2 software (Vigene Tech Inc., Carlisle, MA, USA). For RPPA analysis antibodies for CD74 and SPINT were from Sigma-Aldrich Ltd (Gillingham, UK); antibodies for HMGB1, TFRC, FPN, MYD88, BMP6, TMPRSS6, and HPX were from Abcam 330 (Cambridge, UK); antibodies for STAT5 and JAK2 were from Cell Signaling (Danvers, MA, USA); antibody for STAT3 was from Millipore (Temecula, CA, USA); antibody for FTL was from Santa Cruz (Santa Cruz Biotechnology Inc., Dallas, TX, USA); and, antibody for INHA was from Thermo Fisher (Thermo Fisher Scientific, Waltham, MA, USA).

### 4.4. Statistical Analysis

The univariate comparison of the expression of each protein measured with ELISA or RPPA between benign and malignant tissue was firstly performed by resorting to the non-parametric Wilcoxon–Mann–Whitney test, as well as the comparisons between ER positive vs. ER negative patients [35]. Subsequently, a logistic regression model [36], including age as a covariate, was implemented for each of the proteins selected according to the non-parametric test. In such analysis the relationship between each protein and the breast tissue type was investigated by resorting to a regression model based on restricted cubic splines [37] and, for all of the considered proteins, a linear relationship was observed. To consider the simultaneous determination of the iron-related proteins with RPPA, the *p*-value of the specific test was adjusted according to the Bonferroni method. In addition, in order to evaluate if the expression of the 17 iron-related proteins jointly considered was able to discriminate between benign and malignant tissue, we resort to the linear discriminant analysis (LDA) [38]. This multivariate technique provides a linear combination (i.e., canonical correlation) of proteins expression that maximizes the separation between the two compared groups under the assumption of a multivariate normal distribution within each group and by considering a common covariance matrix. The SMD between group means was used in order to describe how the exclusion of one protein at time influences the capability of discriminate between cancerous and non-cancerous tissue. The SMD was computed by using a pooled covariance matrix. Finally, according to the leave one out cross-validation method, each observation of the dataset was classified by using the discriminant function computed by considering the selected proteins excluding the observation being classified.

All statistical analyses were carried out with the SAS software (Version 9.2.; SAS Institute Inc., Cary, NC, USA). The considered significance level was α = 0.05.

## 5. Conclusions

In this study, we have found an association between the expression of the follow proteins (STAT5, STAT3_pS727, BMP6, CD74, TFRC, INHA, and STAT5_pY694) involved in iron homeostasis and the type of tumor tissue (breast cancerous vs. non-cancerous). Additionally, the multivariate analysis yielded a classifier of 10 proteins that effectively separated the non-cancerous from cancerous tumors.

## Figures and Tables

**Table 1:** Descriptive statistics for the proteins evaluated by enzyme-linked immunosorbent assay (ELISA) according to the tumor lesion.

Proteins	Tumor Lesion	*N*	Minimum	Median	Maximum	IQR ^1^
Hepcidin (ng/mL)	non-cancerous	19	0.002	0.60	8.40	1.58
	cancerous	52	0.03	1.75	48.97	3.00
IL-6 (pg/mL)	non-cancerous	19	1.01	2.15	56.70	1.84
	cancerous	50	1.01	2.32	27.64	2.98
EPO (pg/mL)	non-cancerous	24	6.04	18.29	45.48	18.65
	cancerous	56	5.01	19.78	55.35	9.92

^1^ IQR: Interquartile range; IL-6: Interleukin-6; EPO: Erythropoietin.

**Table 2:** Descriptive statistics of the 17 iron-related proteins measured by reverse phase protein array (RPPA) (arbitrary units) according to the tumor lesion.

Proteins	Tumor Lesion	*N*	Minimum	Median	Maximum	IQR ^1^
BMP6	non-cancerous	24	12,259.0	28,427.5	52,052	10,662.5
	cancerous	56	9672.0	20,090.5	40,135	5800.0
INHA	non-cancerous	24	20.9	16,370.5	106,938	54,852.5
	cancerous	56	7016.0	58,689.0	113,550	39,665.5
FTL	non-cancerous	24	3558.0	5547.0	108,012	18,734.5
	cancerous	56	2492.0	7292.0	67,508	15,029.0
FTH	non-cancerous	24	2922.0	59,107.5	140,084	70,455.0
	cancerous	56	15,093.0	59,281.5	120,572	21,168.0
CD74	non-cancerous	24	5843.0	45,121.5	80,822	34,907.0
	cancerous	56	21,547.0	75,358.0	112,420	29,071.0
FPN	non-cancerous	24	11,980.0	21,205.0	30,946	5768.0
	cancerous	56	12,432.0	24,588.0	40,538	7937.5
HPX	non-cancerous	24	54,721.0	87,553.0	113,550	13,154.5
	cancerous	56	48,050.0	84,120.0	134,592	19,981.0
HMGB1	non-cancerous	24	2592.0	21,306.5	70,969	29,248.0
	cancerous	56	5693.0	33,034.5	73,130	23,245.5
TMPRSS6	non-cancerous	24	561.0	2135.0	63,577	16,252.0
	cancerous	56	670.0	1686.5	83,283	2000.5
MYD88	non-cancerous	24	4949.0	7820.5	14,343	2280.5
	cancerous	56	3569.0	6995.0	27,174	2887.0
JAK2	non-cancerous	24	12,965.0	22,371.5	33,860	6968.0
	cancerous	56	11,790.0	25,085.5	48,533	9799.0
SPINT1	non-cancerous	24	180.0	5993.0	66,171	17,960.0
	cancerous	56	632.0	5913.5	43,045	11,015.0
STAT3	non-cancerous	24	605.0	1685.5	7087	1643.5
	cancerous	56	1779.0	6741.5	43,914	5621.5
STAT3_pS727	non-cancerous	24	2492.0	5564.0	14,400	3766.5
	cancerous	56	3548.0	7009.0	40,538	3507.5
STAT5	non-cancerous	24	7208.0	20,465.5	30,946	7034.5
	cancerous	56	5745.0	8304.0	25,336	5038.0
STAT5_pY694	non-cancerous	24	2055.0	3554.5	5625	1425.5
	cancerous	56	2660.0	4806.0	23,861	3023.5
TFRC	non-cancerous	24	317.0	4293.5	34,892	7021.5
	cancerous	56	2954.0	16,863.5	81,634	23,502.0

^1^ IQR, interquartile range. BMP6, bone morphogenetic protein 6; INHA, inhibin A; FTL, ferritin light chain; FTH, ferritin heavy chain; CD74, cluster of differentiation 74; FPN, ferroportin; HPX, hemopexin; HMGB1, high-mobility group box 1; TMPRSS6, transmembrane protease, serine 6; MYD88, myeloid differentiation primary response gene 88; JAK2, Janus kinase 2; SPINT1, serine peptidase inhibitor, Kunitz type 1; STAT3, signal transducer and activator of transcription 3; STAT5, signal transducer and activator of transcription 5; TFRC, transferrin receptor.

## Supplementary Materials

Supplementary materials can be found at www.mdpi.com/1422-0067/18/2/410/s1.
