# Supplementary material for: Expression of Iron-Related Proteins Differentiate Non-Cancerous and Cancerous Breast Tumors
Source: Int J Mol Sci. 2017 Feb 14;18(2):410. doi: 10.3390/ijms18020410 (PMC5343944; doi:10.3390/ijms18020410)
Supplement: Supplementary file 1 [file ijms-18-00410-s001.pdf]

Supplementary Materials: Expression of Iron-Related Proteins Differentiates Non-Cancerous and Cancerous Breast Tumors

Sara Pizzamiglio, Maida De Bortoli, Elena Taverna, Michele Signore, Silvia Veneroni, William Chi-shing Cho, Rosaria Orlandi, Paolo Verderio and Italia Bongarzone

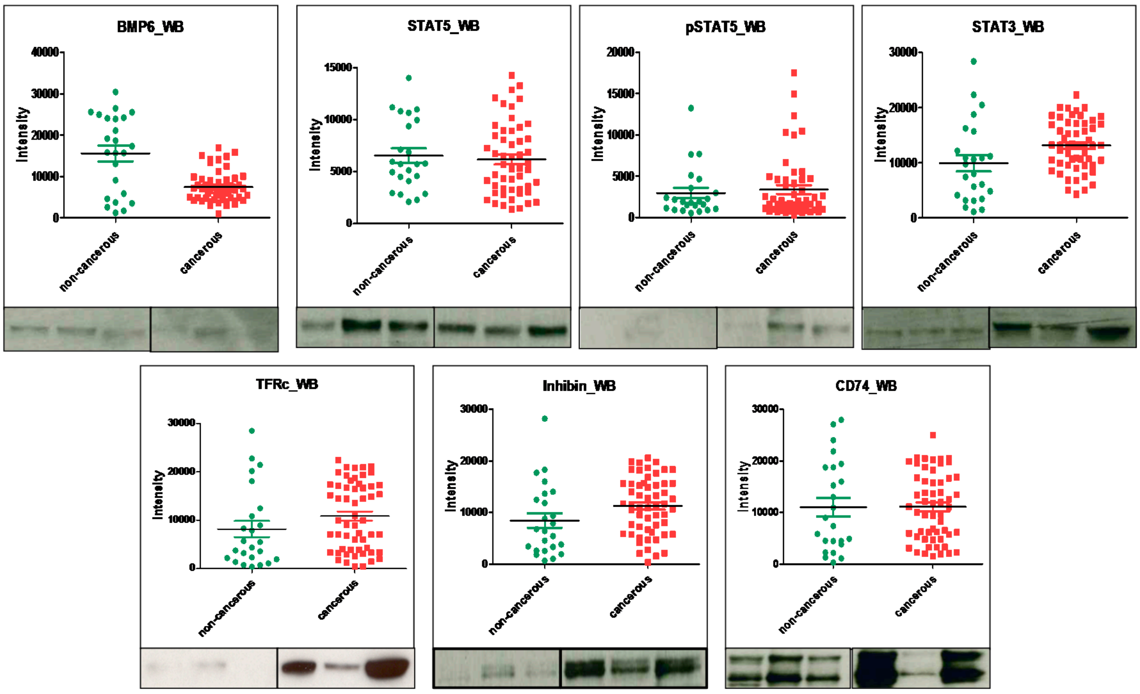

Figure S1. Plots of the individual data intensities obtained in Western blot experiments; Western blotting results probing non-cancerous and cancerous tissue lysates with antibodies for BMP6, STAT5, STAT5_pY694, STAT3, CD74, TFRC, Inhibin, and CD74; Immunoreactive bands were visualized using horseradish peroxidase–linked anti-mouse, anti-rabbit, or anti-goat antiserum and detected using an enhanced chemiluminescence system (Bio-Rad Laboratories, Milan, Italy); Protein quantification was performed using Image J 1.38Xsoftware (http://rsbweb.nih.gov/ij/index.html). Each point represents signal intensity; Black horizontal bars indicate the mean of signal intensities; Plots were obtained using GraphPad Prism 4.0 (GraphPad Software, Inc., San Diego, CA, USA).

Table S1. Clinical-pathological characteristics of the 56 malignant breast tumors.

Sample	Age	ER	PgR	Histology	HER2_ Image J	Arbitrary Evaluation
1	54	positive	positive	invasive ductal carcinoma	1066	negative
2	64	positive	positive	invasive ductal carcinoma	604	negative
3	42	negative	negative	invasive ductal carcinoma	455	negative
4	44	positive	positive	mixed	758	negative
5	51	positive	positive	invasive ductal carcinoma	516	negative
6	not available	not available	not available	not available	890	negative
7	35	negative	negative	invasive ductal carcinoma	597	negative
8	59	positive	positive	invasive ductal carcinoma	850	negative
9	68	positive	positive	mixed	1143	negative
10	64	positive	positive	invasive ductal carcinoma	2323	possibly positive
11	51	negative	negative	mixed	11436	unequivocally positive
12	45	positive	negative	invasive lobular carcinoma	1270	negative
13	68	not available	not available	invasive ductal carcinoma	1610	negative
14	59	not available	not available	mixed	1817	negative
15	74	positive	positive	invasive ductal carcinoma	452	negative
16	81	negative	negative	neuroendocrine	740	negative
17	47	positive	positive	invasive ductal carcinoma	1068	negative

Table S1. *Cont.*

Sample	Age	ER	PgR	Histology	HER2_ Image J	Arbitrary Evaluation
18	51	positive	positive	invasive ductal carcinoma	624	negative
19	51	positive	negative	invasive ductal carcinoma	932	negative
20	not available	not available	not available	mixed	1173	negative
21	63	positive	negative	invasive ductal carcinoma	862	negative
22	69	positive	negative	invasive lobular carcinoma	3898	possibly positive
23	44	positive	negative	invasive lobular carcinoma	1183	negative
24	82	not available	not available	invasive lobular carcinoma	1926	negative
25	34	positive	positive	invasive ductal carcinoma	669	negative
26	67	positive	positive	invasive lobular carcinoma	657	negative
27	not available	not available	not available	not available	1111	negative
28	61	positive	positive	invasive ductal carcinoma	1042	negative
29	52	negative	negative	invasive ductal carcinoma	1145	negative
30	55	negative	negative	invasive ductal carcinoma	13684	unequivocally positive
31	62	positive	negative	invasive lobular carcinoma	627	negative
32	67	positive	positive	invasive ductal carcinoma	981	negative
33	43	negative	negative	invasive ductal carcinoma	675	negative
34	49	negative	negative	invasive ductal carcinoma	803	negative
35	67	positive	positive	invasive ductal carcinoma	2640	possibly positive
36	not available	positive	negative	not available	434	negative
37	58	positive	positive	invasive ductal carcinoma	1636	negative
38	50	positive	positive	invasive ductal carcinoma	2042	possibly positive
39	74	positive	positive	invasive ductal carcinoma	11977	unequivocally positive
40	49	negative	negative	invasive ductal carcinoma	1295	negative
41	59	positive	positive	invasive ductal carcinoma	1178	negative
42	50	negative	negative	invasive lobular carcinoma	9827	unequivocally positive
43	66	not available	not available	invasive ductal carcinoma	2470	possibly positive
44	57	negative	negative	invasive ductal carcinoma	8334	unequivocally positive
45	29	negative	negative	invasive ductal carcinoma	7464	possibly positive
46	74	negative	negative	invasive ductal carcinoma	1159	negative
47	46	positive	positive	invasive ductal carcinoma	1356	negative
48	51	positive	positive	invasive ductal carcinoma	6317	possibly positive
49	40	negative	negative	invasive ductal carcinoma	1485	negative
50	64	positive	positive	invasive ductal carcinoma	1484	negative
51	66	negative	negative	invasive ductal carcinoma	14510	unequivocally positive
52	66	positive	positive	invasive ductal carcinoma	2239	possibly positive
53	not available	positive	positive	invasive lobular carcinoma	1522	negative
54	47	negative	negative	invasive ductal carcinoma	2104	possibly positive
55	not available	not available	not available	not available	621	negative
56	70	not available	not available	invasive lobular carcinoma	346	negative

Except for 4 cancerous samples for which samples were immersed in a protein extraction solution at the moment of receipt and tissue sections were not available, all histology was examined by experienced pathologists at Fondazione IRCCS Istituto Nazionale dei Tumori of Milan; Cases were consecutively collected in the period 1990–1993 at Fondazione IRCCS Istituto Nazionale Tumori of Milan and the Table reports the available clinical-pathological characteristics of the 56 patients with malignant tumor. * Note that HER2 scoring criteria were based on Western blot signal intensity obtained by densitometric analysis with image J software 1.38X (http://rsbweb.nih.gov/ij/index.html); This results may have value for research purposes only; ER: estrogen receptor; PgR: progesterone receptor; HER2: human epidermal growth factor receptor 2.

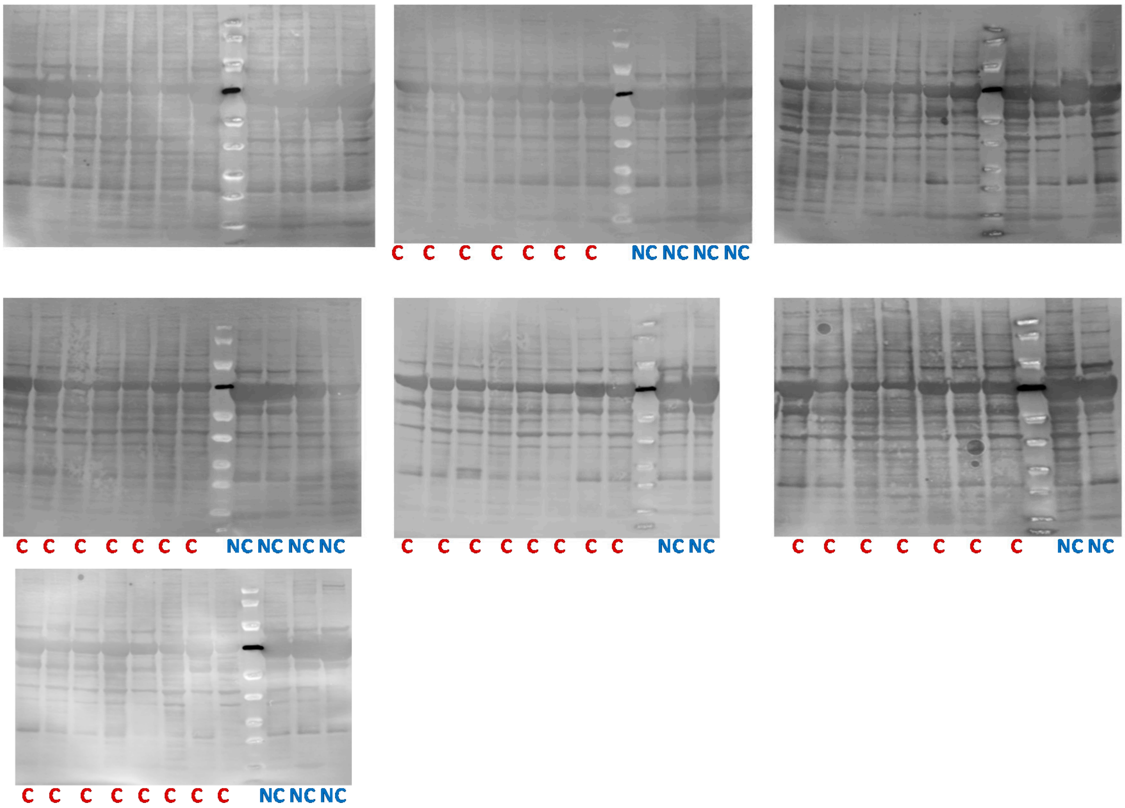

Figure S2. SYPRO Ruby staining of Western blots; Quality control of proteins derived from tissue samples; SYPRO Ruby protein blot stain (Molecular Probes) were used for protein quality assessment; Profile heterogeneity of C and NC samples was interpreted as variation caused by heterogeneity of samples in most respects, including its cellularity, genetic, hystotype and biology; C: cancerous sample; NC: non-cancerous sample.

SUPPLEMENTARY EXPERIMENTAL PROCEDURES

Western blot staining with SYPRO Ruby

All electrophoresis (SDS-PAGE) and electroblotting experiments were performed with precast polyacrylamide NuPAGE NOVEX gels (Invitrogen, Milan, Italy) and with Hybond-C super nitrocellulose membrane (Amersham Biosciences, Little Chalfont, UK).

Proteins were transferred to nitrocellulose membranes, then stained with SYPRO Ruby according to the manufacturer's instructions.

For Western blot analysis antibodies for CD74 was from Sigma-Aldrich Ltd (The Old Brickyard New Road Gillingham Dorset SP8 4XT); antibodies for TFRC and BMP6 were from Abcam 330 (Cambridge, UK); antibodies for STAT5 were from Cell Signaling (Danvers, MA, United States); antibody for STAT3 was from Millipore (Temecula, CA, USA); antibody for INHA was from Thermo Fisher (Thermo Fisher Scientific, Waltham, MA, USA); and antibody for HER2 antibody (Ab3) was purchased from CalBiochem/Oncogene Research Products (Cambridge, MA, USA).
